# The Significance of Persistory Pyrexia

**Published:** 1907-11-23

**Authors:** 


### The Hospital

A Journal of

The Medical Sciences and Hospital Administration.

New Series. No. 35, Vol. II. [No. 1107, Vol. XLIII.] SATURDAY, NOVEMBER 23, 1907.

### The Significance of Persistent Pyrexia.

The diagnostic problem of a more or less persistent febrile temperature for which no adequate explanation, in the shape of physical signs or otherwise, can be discovered, is one which is not infrequently to be numbered among the anxieties of medical practice. Apart from the burden of uncertainty and suspicion which such circumstances necessarily place on the mind of the practitioner himself, there is the practical difficulty created by the insistent demand of the patient and his friends for a prompt and confident explanation of the condition. Successfully to postpone such a demand is not an easy task, while prematurely to gratify it carries the risk of penalties which sooner or later are inevitably exacted from those who argue in advance of the facts. It may be anticipated that the difficulty here alluded to will be lessened, at least in some degree, as the lay public are educated to appreciate the essential nature of medical methods. Education, however, is a slow process, and meanwhile the situation presents its urgent claim. With that claim it is impossible not to sympathise. Early diagnosis means to the practitioner both successful application of his art and relief from the anxiety to which uncertainty necessarily gives rise. To the patient and his relatives it carries the consolation that the position is at least fully understood, and that the resources of art can therefore be reasonably and confidently directed towards its amelioration. Hence any measure by means of which an early interpretation of an existing febrile disturbance in the individual patient is rendered possible, is to be welcomed, not merely as a scientific advance, but also as an addition to the beneficent range of medical practice. Thus, it appears, there are urgent claims why particular attention should be paid to all possible means which will secure the early recognition and differentiation of diseases characterised by abnormal temperature records. The profession has not been slow to appreciate any advances in this direction, and definite help has been afforded by, for example, Koplik's spots in the diagnosis of measles and the Widal reaction in the recognition of enteric fever. More recently the description of Calmette's serum reaction affords some ground for hope that the early diagnosis, or exclusion, of tuberculosis will be facilitated. Even though all these are recognised, however, there still remain many difficulties in the practical sphere, and these, in the meantime, can only be met by recalling possibilities and thus avoiding any oversight in the attempt at diagnosis.

The early hours or days of a febrile temperature leave the diagnosis open between a "simple febricula" and one or other of the specific fevers, and anxiety hardly arises until by the lapse of time and the non-appearance of any characteristic eruption the latter are excluded from the discussion. It is when a week or more has passed without anything to explain the fever that the case takes on an anxious note, and as the days pass and no interpretation appears the strain of the situation naturally increases. Probably the first question which arises in these circumstances is whether the case may not be an example of enteric fever. Among the various forms which this disease assumes, certainly one may be a mere disturbance of the temperature. Hence it ought to be an invariable rule that any febrile process persisting for eight days or more without obvious explanation demands the application of the Widal test, and with a negative result the test should be repeated. On the other hand, a positive result may be of little value if at some previous date the patient has been the victim of enteric fever. As an alternative to enteric fever, the diagnosis of tubercle has to be considered in the circumstances now under discussion. There is no doubt that this may exist even over a period of many weeks, and as an active disease, without the production of physical signs. And here it is that Calmette's tuberculin reaction would appear to promise help. There is, however, this reflection to be borne in mind. Calmette's test, it is claimed, will produce a positive result so long as tubercle exists anywhere in the body, and this whether the tubercle is active or latent. Now according to the pathologists some fifty per cent. of all bodies examined after death show evidences of tubercle in one or other form. Hence a response to Calmette's test in any individual case is not a proof that an existing febrile disturbance in such a case depends on active tuberculosis, but is only evidence that there is tubercle somewhere or other in the patient's body; and as this conclusion must apparently be accepted of half the patients that come under review, the value of the test would necessarily appear to be limited. The very completeness of its range limits the confidence with which its results can be applied to an actual emergency. And a yet more extended experience is necessary before its limitations and exact value can confidently be defined.

In addition to enteric fever and tubercle, a septic process is necessarily considered in any case of persisting pyrexia. Here the difficulty is to trace the source of the poisoning, for short of this a positive diagnosis is hardly possible. Even when a process of this order produces malignant endocarditis there may be neither valvular murmurs nor embolic phenomena to point to the true nature of the case. Last of all may be mentioned certain blood diseases, especially pernicious anæmia, as events which may give rise to much fever without accompanying physical signs. This possibility should always be borne in mind in the case of febrile disturbance existing without obvious explanation.

